# Cases of Disease in the Urinary Organs

**Published:** 1827-05

**Authors:** 

**Affiliations:** St. George's Hospital


### DISEASES OF THE URINARY ORGANS.

### *Cases of Disease in the Urinary Organs.* Treated at St. George's Hospital, by Mr. Jeffreys.

Case I. *An Account of some unusual Morbid Appearances found in the Kidney of a Man, who died in* St. George's Hospital.

In the following case we have an example of the extent to which morbid alteration of structure may sometimes proceed, without any suspicion of its existence being excited, during life, in the mind either of the patient or his medical attendant. It is impossible to guess how long the disease in the kidney had existed; but it is to be presumed that the patient suffered no inconvenience from it as far as regarded the secretion of urine, since the experiments of physiologists have proved that one of the kidneys may be removed from a living animal with impunity.

John Cook, aged fifty-one, became an out-patient at St. George's Hospital, in the beginning of October, 1826, on account of a morbid enlargement of the left testicle, accompanied by a disturbed state of his general health, and anasarcous swellings of his lower extremities. He had been a schoolmaster, and attributed the origin of his complaint to a severe kick from a boy's foot, whom he was chastising about a year ago. This was followed by considerable swelling and inflammation of the part, for which he underwent the usual mode of treatment; but the testicle remained enlarged and indurated, and he then went through a long course of mercurial inunction, and took sarsaparilla, under the direction of the late Mr. Pearson.

At the time he became an out-patient at the hospital, the testicle was larger than a hen's egg, flattened at its sides, and hard and knotty; but the induration was chiefly confined to the epidydimis, which was painful, and irregular in its shape: the chord was not affected. Towards the end of November, he was attacked with diarrhœa, which proved obstinate and intractable; the anasarca of the leg increased; and, as his means of subsistence were unequal to the necessities of his situation, he was admitted an in-patient on the 13th of December. Nothing, however, checked the constant purging, and the anasarca spread over the whole body. On the 26th, he was seized with stupor and paralysis of the right side; and on the 3d of January he died.

His urine had been scanty and high coloured, but in other respects healthy; and he never complained of any particular pain or uneasiness about the loins.

*Dissection.*—No morbid appearances were observed in the head, except that the lateral ventricles of the brain contained altogether about half an ounce of water.

Serous effusion to some extent had taken place in each side of the thorax, but the viscera of these cavities were free from disease.

The following curious appearances were observed in the left kidney:—It had no vestige remaining of its natural structure; the pelvis and infundibula were entirely obliterated, and had assumed the appearance of a membrane without any cavity. The ureter, for two-thirds of its length, was also obliterated, and had become a mere cord. The body of the kidney, which was nearly of its natural size, was made up of a number of cysts, some of which were as large as a filbert. They had no communication with each other, and contained a peculiar kind of chalky looking matter, which was in some of the cysts nearly solid, and in others in a semi-fluid state, like soft mortar. Some of this substance was analysed by Dr. Prout, and found to consist of "phosphate of lime, with animal matter." The opposite kidney was somewhat larger than natural, but its structure appeared to be healthy.

The other viscera of the abdomen were in a sound state.

The left testicle was of the size of a hen's egg. When cut into, a multitude of small white granular bodies, apparently approaching to cartilaginous structure, were found interspersed amongst the tubuli testis. They were so numerous, that a good deal of glandular structure seemed to be completely destroyed. The epidydimis was considerably enlarged, and contained several small cysts full of a semi-fluid substance, of a yellow cheesy colour. This substance, when examined by Dr. Prout, was found to contain "little or no earthy matter, and to be merely a peculiar form of albumen." The right testicle was but little increased in size, and its glandular structure appeared to be in a healthy state; but the epidydimis was enlarged, and some cysts were formed in it containing a white, half-fluid substance.

The bladder was considerably dilated, and its coats were thinner than natural. The prostate gland was much larger than it ought to be, and contained a number of calculi imbedded in separate cysts. According to Dr. Prout's analysis, the composition of these substances was similar to that contained in the left testicle.

These specimens of morbid structure are preserved in Mr. Brodie's Museum in Great Windmill-street.

Case II. *Calculi and Ulceration in the Kidneys.*

One result of the long-continued residence of calculi in the kidney, especially if they are large and rough, is suppuration and ulceration of its glandular structure, denoted by increase of pain, irritation, and hectic, and the expulsion of pus, and occasionally blood, with the urine. The sufferings of the patient are often truly deplorable, and terminate only with his existence. Upon examination after death, as in the following instance, masses of calculous matter are found filling up the cavities of the gland, and more or less of its substance is destroyed by ulceration.

James M'Kenvey, a poor Irishman, was brought to St. George's Hospital, April 1st, 1824, in a very weak and exhausted condition, and apparently dying from the joint effects of disease and want. He complained of great pain in the back, and about the pubes and bladder, and was tormented with an incessant irritation and difficulty in making water, which was voided in a small quantity at a time, and loaded with blood and purulent matter. He had a brown, dry tongue; a quick, feeble pulse; and his bowels had not been relieved for some days.

It appeared, from the little information he was able to give of himself, that he had undergone the operation for stone about ten years before: since then he had been in the habit of passing small calculi from time to time by the urethra; and, about a fortnight before his admission, his urine had, for the first time, become tinged with blood, and within the last four days loaded with pus.

The house-surgeon gave him a dose of Castor-oil, and had him put into a warm bath; after which a catheter was introduced into the bladder, and about two ounces of exceedingly offensive purulent urine were drawn off. Plenty of nourishing food, with brandy, &c. was directed for him; but he continued to sink, and on the following day at noon he died.

On examining the body after death, both kidneys were found to be larger than natural; and, when cut into, the pelvis and infundibula of each were filled with masses of calculous matter, moulded to the size and shape of those cavities, and surrounded with purulent matter. These masses were of considerable size, and the pressure of their rough and uneven surfaces had excited extensive ulceration and destruction of the secreting structure in both kidneys. The ureters were slightly enlarged, and the coats of the bladder thicker than natural.

Case III. *Stricture in the Urethra, with Swelling of the Prostate Gland, and a large Abscess between the Abdominal Muscles and the Peritoneum.*

Thomas Smith, fifty-seven years of age, was admitted into the hospital, April 5th, 1824, with a frequency and difficulty in making water. He was then in a state much resembling that of low fever; was restless and uneasy; had a dry, brown tongue; and was very low and weak. He said that, seventeen years ago, he was cured in this hospital of strictures in the urethra, under Sir Everard Home; that he had been suffering for several weeks with his present complaints, and had been attended by a surgeon, who introduced the catheter almost daily, but without affording much relief to his symptoms, and had therefore advised him to come to the hospital.

At this time there was a large tumor situated under the abdominal muscles, which reached nearly from the pubes to the navel, was distinctly circumscribed, and very much resembled in its shape and appearance a dilated bladder; but it was hard and incompressible, giving to the fingers rather the feel of a solid structure, and very painful and tender when pressed or examined. Upon introducing a silver catheter into the urethra, it passed with perfect ease up to the rings, and appeared to go into the bladder; but only a little fetid blood, and no urine, flowed out, the instrument having slipped into a false passage. On examining with the finger in ano, it was ascertained that there was a considerable enlargement of the prostate gland. A flexible gum catheter was then passed, which, with a little management, entered the bladder, and three ounces of fetid urine were drawn off. But this was not followed by any relief from pain, nor by any diminution in the size of the tumor.

Twelve leeches were directed to be applied to the region of the pubes. He was put into the warm bath, and had fifteen grains of Dover's powder at bed-time; and the catheter was introduced three or four times a-day, or as often as he had an inclination to empty the bladder.

Under this treatment his urine became clear and natural; and, on one occasion, as much as three pints were drawn off, but without making the slightest alteration in the size or appearance of the swelling above the pubes. Sometimes the instrument would slip into the false passage, and, when this happened, nothing passed out but a small quantity of offensive blood, mixed with an oily kind of matter, not at all resembling pus. By degrees the tumor became more soft and flabby, and a kind of deep-seated indistinct fluctuation could be perceived in it. His debility and exhaustion increased daily; he fell into a stupid, comatose state; had a dry, brown tongue; a quick, feeble pulse; would take very little nourishment; and he sunk, and died on the 15th, ten days after his admission. During the last few days of his existence, not more than two ounces of urine were drawn off at a time.

When the body was examined the next day, there was found a large cyst or abscess in the cellular structure between the peritoneum and the muscles of the abdomen, which extended from behind the pubes nearly to the navel, and was capable of containing a pint of fluid. Its internal surface had a dark, greenish-black colour; and in its cavity was about half an ounce, or rather more, of offensive, ill-conditioned matter, mixed with blood, like that already described. The membranous portion of the urethra was thickened and contracted, and in the upper part of it, anterior to the stricture, there was a false opening, or passage, leading up between the neck of the bladder and the pubes, and communicating with the abscess. This rent had evidently been made by misdirected and too-forcible attempts to pass instruments into the bladder. The prostate gland was enlarged to three times its natural size, and the coats of the bladder were thicker than usual.

It is worthy of observation, that there had been no discharge from the urethra either of blood or pus, at least after his admission into the hospital. On a superficial examination, the tumor might have been readily mistaken for a distended bladder, which it closely resembled in shape and situation. But drawing off the urine made no alteration in its size or appearance; which, together with its apparent firmness of structure, and the oily kind of bloody discharge that escaped through the catheter whenever that instrument got into the false opening in the urethra, induced a suspicion of the tumor being of a fungoid character.

The case affords a striking lesson of the dangerous consequences that may arise from using force and violence in the attempt to introduce catheters and other instruments into the bladder. It was altogether inexcusable in this instance; for there was no difficulty in passing the catheter after its point had been conducted beyond the false opening in the urethra.

Case IV. *Diseased Prostate, with Dilatation of the Bladder, and Enlargement of its Muscular Fibres.*

—— Bricard, a feeble old man, seventy-four years of age, came under my care May 5th, 1824, on account of a difficulty in making water, which he said he had been troubled with for eight months, or more. He had an almost constant desire to void his urine, but could only evacuate a few ounces at a time. He had been some time in the hospital under the care of the physician, and the house surgeon had been in the habit of drawing off his water twice a-day, from one to three pints at a time. On examining the bladder with the finger in ano, the prostate gland was found to be considerably enlarged; but there was no difficulty in introducing a catheter into the bladder, and, ten minutes after he had made water by his own efforts, twenty-eight ounces more were drawn off by means of that instrument.

He was ordered to go into the warm bath occasionally; to take five grains of the Extract of Conium, and as much of the Plummer's Pill every night at bed-time; and to have his water drawn off three times a-day.

But the quantity of urine which remained in the bladder increased, instead of diminishing. On one occasion thirty-two, and upon another forty-two, ounces were drawn off. A flexible gum catheter was therefore placed in the bladder, with a stopper fixed in it, by means of which the patient was enabled to relieve himself whenever the inclination to make water came on, and the bladder was thus kept comparatively empty.

Still the secretion of urine continued very abundant, and he did not recover the power of emptying the bladder by his own efforts. The continued residence of the catheter in the bladder at length began to occasion great pain; and, on the 27th of June, his water became tinged with blood. For these reasons the instrument was taken out, and passed four or five times in the twenty-four hours, instead of being left constantly in the bladder. This operation excited exquisite pain at the neck of that viscus: pus and mucus, as well as blood, came away with his urine. He grew daily more low and weak; and had a quick, feeble pulse, and a dry, parched tongue. His water also became very offensive, as well as loaded with mucus: the last drops were tinged with blood, and were voided with great pain at the neck of the bladder. The warm bath, Dover's Powder, Liquor Potassæ, Laudanum, &c. appeared to afford but little, and only transitory, relief. He continued gradually growing worse until the 7th of July, when the powers of life seemed to be quite overcome, and on the following morning he died.

The next day the body was examined. The coats of the bladder were preternaturally thickened, in some places to the extent of half an inch. Its muscular fibres were much larger and more distinct than usual, and between their fasciculi the inner coat was thrown into pouches large enough to receive the end of the finger. All the lobes of the prostate were considerably enlarged; and the middle lobe had two nipple-like processes projecting into the cavity of the bladder. The point of one of these was in a state of ulceration, which sufficiently accounted for all the pain the poor man had endured every time the catheter was passed, or whenever he made water; as well as for the discharge of blood. No morbid appearances were observed in the ureters or kidneys, except that the pelves of the latter were rather larger than usual.

CASE V. *Abscess in Perineo, with old Stricture in the Urethra.*

John Peglar, sixty years of age, was admitted into St. George's Hospital, April 7th, 1825, having a large phlegmonous abscess in the perineum, and an œdematous swelling of the penis and scrotum, which had acquired the size of an ostrich's egg. A distinct fluctuation could be felt in the abscess, which filled up the whole of the perineum; and the skin over it was of a deep red colour: but that covering the penis and scrotum was paler, and the swelling had a firm, semi-pellucid appearance, without any feeling of crepitus or emphysema when examined. He was suffering great pain in these parts, especially in the perineum; had an anxious, distressed countenance; a great deal of fever; a dry, furred tongue; and a very quick, irregular pulse.

It appeared that he had been subject to bad strictures in the urethra for many years; that he had been in the habit of passing bougies for himself, and had at different times experienced very alarming attacks of retention of urine. His present symptoms, he said, came on five days before, with swelling and pain in the perineum, attended with rigors and much fever; but without any stoppage in his water, which, he assured me, had been voided daily in a stream up to the time of his admission.

I immediately made an incision with a scalpel through the whole extent of the abscess in perineo, and let out full three ounces of offensive pus and urine, much to the relief of the poor man's sufferings. He was then directed to have the parts well fomented; to take a dose of Castor-oil; to go into the warm hip-bath as soon as it could be got ready; and to have ten grains of Dover's powder at bedtime.

From the truly circumscribed character of the abscess, and the tumefaction of the penis and scrotum being entirely devoid of the boggy emphysematous feel which commonly attends extravasation of urine into those parts, I considered the swelling to arise merely from serous infiltration into the cellular structure, in consequence of the inflammatory action having extended itself from the perineum; and that it would all subside as soon as a free vent should be given to the contents of the abscess. I, therefore, refrained from making any scarifications through the skin of the penis and scrotum; and, as the opening made in the perineum was sufficient to allow of the ready discharge of any urine that might escape from the urethra, I determined on waiting until the inflammation and swelling of the parts should have subsided, before I attempted to pass any instrument into the bladder. In the evening, however, the house-surgeon introduced a small flexible gum catheter into the bladder, where he left it.

The man passed a tolerably comfortable night; and on the following day, April 8th, the swelling of the penis and scrotum, together with the febrile symptoms and pain, were greatly reduced, and he was in all respects much relieved. The catheter was therefore allowed to remain in the bladder; but it excited uneasiness in the urethra, and on the evening of the 10th the pain and irritation it occasioned were so great, that he could bear it no longer, and he took out the instrument. In the mean time, the wound in the perineum assumed a healing appearance, the swelling of the penis and scrotum continued to subside, and by the 13th that and the fever had entirely disappeared. He could now make water in a stream, and without much difficulty, through the urethra. Nevertheless, the wound did not completely heal: a fistulous sinus remained, through which a few drops of urine occasionally escaped. I endeavoured to remedy this by dilating the strictures, the chief of which was found at the membranous part of the canal, but could only succeed in getting a bougie or a flexible gum catheter, of less than the middle size, into the bladder, in consequence of the external orifice of the urethra being very much contracted from original malformation. When the catheter was left in the bladder, if it was only for an hour, irritation and dysury were sure to be brought on, and the urine was forced out by the wound and along the sides of the instrument. It was, therefore, only introduced every second or third day; and these effects did not follow when it was simply passed and withdrawn. Now and then the catheter would slip into a false passage; but, by giving it a particular curve, and passing it with a good deal of care, I was enabled in general to avoid this accident.

By degrees the size of the catheter was increased, and, towards the beginning of August, one above the middle size could be introduced with tolerable facility; and he made water with more freedom and comfort, and was able to retain it longer than for many years. Several days would now pass without any urine escaping by the fistula in the perineum. Still the sinus did not entirely close up, and a very small quantity of matter oozed from it and the urethra.

During his residence in the hospital, he had had three or four severe attacks of gout in the larger joints of both the upper and lower extremities, a disease to which he had been many years subject; and his health was beginning to suffer from the confinement and air of the house. On this account he begged to be made an out-patient, and he was discharged as such on the 17th of August, with directions to attend twice or three times a-week, to have the catheter passed. But he paid little attention to this injunction, having been in the habit, as has been stated, of passing bougies and other instruments for himself. He came two or three times to the hospital during the first fortnight; but after that I saw no more of him till the 15th of the following December, when he was readmitted under the care of Dr. Hewett, in a very reduced and exhausted state, labouring under chronic diarrhœa, cough, and profuse purulent expectoration. These symptoms gradually undermined the powers of his constitution, and he sunk, and died on the 4th of March following.

When readmitted, there was a small fistulous orifice still remaining in the perineum, just large enough to admit a pin: through this a few drops of urine occasionally passed when he made water, but he was able to void it in a tolerably free stream by the urethra, having continued to pass the bougie or catheter for himself since he quitted the hospital, although he had seldom been able to get it beyond the stricture at the membranous part. This was, however, sufficient to keep him comparatively comfortable in that respect; and he was allowed, at his particular desire, to go on in his own way, as far as regarded the management of his strictures.

The following appearances presented themselves at the examination of the body:—Almost universal adhesion of the right lung to the pleura costalis; large tubercles and vomicæ in the upper part of each lobe, and the branches of the bronchi full of pus. Upon the mucous membrane of the caput coli, and the great arch of the colon, were numerous ulcerations, irregular in shape, and varying in size from a large pin's head to a half-crown.

The bladder and penis were removed from the body, and the urethra laid open its whole extent. The meatus externus was much contracted from original malformation. At the membranous portion was a permanent stricture, through which a common probe could with difficulty be passed: in front of this was a false opening, which communicated with a cavity about half an inch in diameter, running backwards for about an inch and a half towards the neck of the bladder. This cavity had a smooth even surface, and at its posterior extremity, next the bladder, there was a small opening, which proved to be the termination of the fistulous sinus in the perineum. The bladder and prostate were not diseased.

Case VI. *Suppression of Urine, and Death, after a Blow on the Perineum.*

John Gay, about twenty-two years of age, on the evening of Sunday, 17th July, 1825, received a violent kick on the perineum from a man's foot. A profuse hemorrhage immediately took place from the urethra, and he nearly fainted from the excessive pain which the blow occasioned. On the following morning, the 18th, he was brought to the hospital, having been unable to make water since the accident, and suffering much uneasiness from the distention of his bladder. There was a good deal of discoloration about the perineum, from extravasation of blood; but very little swelling, and not so much tenderness as might have been expected. The house-surgeon passed a silver catheter into the bladder, and drew off a pint and half of urine, mixed with dark-coloured blood. The hemorrhage had previously ceased, and there was now only a slight oozing of blood from the urethra.

He was directed to have some house-physic; to keep the contused parts constantly wet with compresses soaked in cold spirit lotion; and a flexible gum catheter was placed in the bladder, in order to prevent any effusion of urine from taking place through the ruptured portion of the urethra.

In the evening, it was found that no water had been voided through the catheter, the eyes of it having been blocked up with coagulated blood; and there was a good deal of pain and distention about the bladder in consequence. The instrument was therefore taken out, and another introduced, and twelve ounces of urine drawn off.

July 19th.—The water had flowed freely through the catheter during the night; and this morning early he took a Senna draught, which operated well. In the last portions of urine voided to-day were a few drops of blood. He said he was free from pain; he had no fever, and there was scarcely any swelling in the perineum. In the evening, he complained of pain in the head, and had made no water since the middle of the day; he had a dry, furred tongue, and a small, weak pulse. A fresh catheter was passed, but there was no urine found in the bladder, and only a few drops of blood came away.

He was ordered a draught with thirty drops of Laudanum and a drachm of Sp. Æther. Sulph. comp.

20th.—Had a restless night, but did not complain of pain. He had made no water, and was much in the same state as on the preceding evening. The catheter was again passed, but no urine was found in the bladder. There was no particular heat of skin nor thirst, nor urinous smell about his person, as is sometimes the case where the secretion of urine is suppressed.

He was directed to be put into the warm bath, and to take the following draught every two hours:—℞. Pulv. Ipecac. co. gr. v.; Potassæ Nitr. gr. x.; Pulv. Tragæ. co. ʒss.; Aquæ Piment. ℥jss.; Sp. Æth. Nitr. ʒj. M. fiat haustus.

In the evening he became comatose, and the skin over the whole body completely jaundiced; and on the following day, at eleven o'clock, he died. About two hours before he expired, the catheter was passed, and two ounces of dark-coloured offensive urine were drawn off.

At the examination of the body after death, the urethra was found to have been extensively ruptured between the bulb and the prostate gland; and the cellular structure surrounding that part, and in the perineum, was loaded with a profusion of dark-coloured coagulated blood, but without any admixture of pus or urine in it. The bladder was quite empty, and in a perfectly healthy state; nor could any disease be observed in the kidneys, or in any of the viscera of the abdomen. The gall-ducts were not obstructed.

In this case the suppression of urine probably depended on nervous sympathy, or consent, between the kidneys and the urethra. It came on at the expiration of nearly forty-eight hours after the receipt of the accident, at a time when every thing appeared to be going on in the most favourable way, and destroyed the patient at the termination of about forty hours.

A jaundiced state of the skin, such as made its appearance in this case, is not uncommon in persons who die of accidents, or after large operations. I have seen it occur in compound fractures, and after amputation.

